# Family and School Contexts as Predictors of Suicidal Behavior among Adolescents: The Role of Depression and Anxiety

**DOI:** 10.3390/jcm8122066

**Published:** 2019-11-24

**Authors:** Nicolás Ruiz-Robledillo, Rosario Ferrer-Cascales, Natalia Albaladejo-Blázquez, Miriam Sánchez-SanSegundo

**Affiliations:** Department of Health Psychology, Faculty of Health Sciences, University of Alicante, 03690 Alicante, Spain; nicolas.ruiz@ua.es (N.R.-R.); natalia.albaladejo@ua.es (N.A.-B.); miriam.sanchez@ua.es (M.S.-S.)

**Keywords:** suicide, adolescents, family, school, depression, anxiety

## Abstract

Suicide is the highest external cause of death in the majority of developed countries. In adolescents, the prevalence of suicide has increased significantly in recent years, becoming a serious public health concern. The main aim of the present study is to characterize suicidal behavior, and to evaluate the relationship between family and school contexts and suicidal behavior through the possible mediating effects of depression and anxiety. The design of the study is cross-sectional. The sample was composed of 1386 Spanish adolescents from 14 high-schools. Suicidal behavior, anxiety, depression, family function, and school climate were evaluated through self-reported questionnaires. The results obtained show a significant association between adaptive family function and a positive school climate with low levels of depression, anxiety, and suicidal behavior. Mediation analyses revealed a significant full mediating effect of depression on family function and school climate with suicidal behavior. No significant mediating effects were found for anxiety. The results obtained underline the importance of family and school as protective factors for the prevention of suicidal behavior in adolescents, through the main mediating role of depression. Future studies should evaluate the mechanisms underlying the effects of family function and school climate on depression, and therefore, on suicidal behavior in adolescents.

## 1. Introduction

Suicide is a major public health concern worldwide [1]. In Spain, suicide is the highest external cause of death in adults and the third-highest cause of death in the population aged between 15 and 29 years [2]. This is especially worrisome as recent studies have identified an increase in suicidal behavior among adolescents in recent years [3]. For example, in a recent study conducted on 1664 Spanish adolescents, 6.9% of participants exhibited high levels of suicide ideation, and 4% had attempted suicide during the previous year [4]. Similarly, in a sample of 151,191 adolescents from the United States of America, 4.2% reported at least one suicide attempt during the previous year [5].

Due to the severe consequences of suicidal behavior during adolescence, the identification of protective factors that could prevent this in a population should be a priority for public mental health policies. In relation to this, community agents, such as family and schools, have been shown to play a critical role in the prevention of suicidal behavior during adolescence [6,7,8,9,10].

Firstly, regarding family, several studies have analyzed the effects of family function on mental health and suicidal behavior in adolescents [6,9,10]. Family function has been defined as the interactions between—and reactions to—family members [11], and such interactions being dysfunctional has been related to mental health disturbances and higher rates of suicidal behavior during adolescence [6,9,10]. In line with this, it has been demonstrated that adolescents who self-harm perceive significant impairments in family function [12]. Several mechanisms have been proposed to explain the relationship between family dysfunction and suicidal behavior in adolescents. A classical review of studies found that parental psychopathology, such as substance abuse, depression, or antisocial behavior are the most significant predictors of suicide in adolescents [13]. Probably poor parental monitoring, family disruption, or family discord could be the basis of the association between parent psychopathology and suicide [13]. Hence, verbal abuse, low adaptability of the family, and low family support have demonstrated to be directly related to the risk of suicide [13]. Furthermore, besides the possible psychopathology of parents, other characteristics of the family context, such as the lack of family cohesion, disconnection between family members, and disrupted communication, have also demonstrated to be significant predictors of suicide ideation during adolescence [14]. As proposed by authors, it is probable that this type of family context promotes feelings of loneliness and abandonment in adolescents, increasing mood disturbances, and hence, suicidal behavior [9,15].

Secondly, another of the most important factors directly related to mental health and suicidal behavior in adolescents is the school climate [16]. School climate can be defined as the quality of the interactions between students, teachers, parents, and school staff, reflecting the norms, values, and goals that represent the educational and social missions of the school [17]. School and the relationships that adolescents establish in this context have been shown to be determinants of socioemotional development during adolescence [18,19], and hence, as would be expected, a negative school climate has been linked to higher levels of mental health problems and suicidal behavior in this population [5,8,16]. A recent study conducted with the participation of 15,191 adolescents has found that individuals with a poorer perception of the school climate are more likely to develop suicide ideation [5]. Specifically, when adolescents were asked to state the main causes of suicidal behavior, most identified bullying and peer problems as the principal reasons [5]. In this regard, difficulties in peer interaction in the school context have demonstrated to be the most significant predictor, especially when adolescents exhibit feelings of disconnection and belongingness from the school and peers, and suffer from any harassment [5,16,20]. Thus, bullied adolescents are twice as likely to develop suicidal behavior than their non-victimized counterparts [20]. Accordingly to the results from previous research, a negative school climate characterized by the lack of cooperation between peers, difficulties in integration, and feelings of disconnection in the school context entails a significant risk factor for the development of suicidal behavior [20]. 

While some studies have found a direct association between family functioning and suicidal behavior in adolescents even after controlling for mental health problems [10,21], others studies have identified several factors that are mediators or moderators of this relationship, in particular, negative effects [6,22]. In this regard, adolescent suicide has been closely related to mental health problems, particularly high rates and severity of symptoms of depression [23] and anxiety [24]. On the other hand, although some studies have identified depression and anxiety as mediators in the association between family function and suicidal behavior, to our knowledge, no studies have compared the mediating effects of depression and anxiety on this association. Further, this issue has not been explored when analysing the association between the school climate and suicidal behavior. Understanding the differential effects of depression and anxiety as mediators would advance our comprehension of the mechanisms underlying the association between specific psychopathology and suicidal behavior.

With all this in mind, recalling that suicide in adolescents is a growing public health concern, the present study aims to characterize the prevalence of suicidal behavior among a representative sample of Spanish adolescents. Furthermore, the study is undertaken to explore the possible preventive effects of family function and school climate, previously described as protective factors, on the suicidal behavior of Spanish adolescents. The third aim of the study is to analyze the possible mediating effects of depression and anxiety on the relationship of family function and school climate with suicidal behavior, identifying possible differences in the mediating effects of these two factors.

## 2. Materials and Methods

### 2.1. Measures

#### 2.1.1. Suicidal Behavior

Suicidal behavior was evaluated using the revised form of the Suicidal Behaviors Questionnaire (SBQ-R) [25]. This 4-item questionnaire is aimed at measuring the lifetime prevalence of suicidal thoughts and behaviors, recent suicidality, and future likelihood of a suicide attempt. Total scores provide an indicator of where an individual lies on the continuum of suicidality, with possible scores ranging from 3 to 18. This questionnaire has been found to be an effective research tool among clinical and non-clinical groups, with an alpha reliability ranging from 0.76 to 0.88 [25]. Consistent with previous studies with the SBQ–R, we used scores on Item 1 to define subgroups of suicidal and non-suicidal participants. 

#### 2.1.2. Depression

Depressive symptoms were assessed using the Patient Health Questionnaire-9 (PHQ-9) [26]. The PHQ-9 is a 9-item self-reported questionnaire designed to evaluate the presence of depressive symptoms during the prior two weeks. As a severity measure, scores can range from 0 (absence of depressive symptoms) to 27 (severe depressive symptoms). Each of the nine items can be scored from 0 (not at all) to 3 (nearly every day). The PHQ-9 has proven to be a valid and reliable assessment of depression across many populations [27,28]. 

#### 2.1.3. Anxiety

Anxiety symptoms were analyzed with the Generalized Anxiety Disorder-7 item scale (GAD-7) [29,30]. The GAD-7 is a one-dimensional scale designed to assess the presence of the symptoms of generalized anxiety. It is self-administered, and the total score is calculated by the simple addition of the scores for each item. The scores of all seven items range from 0 (not at all) to 3 (nearly every day), yielding a total score that ranges from 0 to 21. 

#### 2.1.4. Family Function 

Family function was assessed with the Systemic Clinical Outcome and Routine Evaluation-15 (SCORE-15) [31]. This is a valid and reliable self-reported measure consisting of 15 items ranked on a 5-point Likert scale, grouped into three dimensions: Strengths and adaptability, that evaluates individual perceptions of family resources and support; overwhelmed by difficulties, which evaluates family constraints and disruption; and disrupted communication, that assesses the level of family communication. Higher scores in each of the subscales indicate more difficulties in each of the evaluated family function domains. The Spanish adaptation employed in the present study has demonstrated adequate psychometric properties [32].

#### 2.1.5. School Climate 

School climate was evaluated employing a questionnaire from a Spanish national study on social harmony in secondary education [33]. The overall evaluation instrument was developed with the aim of evaluating the quality of school climates in Spanish high-schools and demonstrated adequate psychometric properties [33]. The form which evaluates students’ perception, composed of 25 items ranked on a 4-point Likert scale, was used for this study. From this form, the subscales of satisfaction (8 items), sense of belonging (5 items), integration (3 items), cooperation (3 items), and communication between family and school (6 items) were selected and responses summed to yield a total school climate score. 

### 2.2. Procedure

The present study is a part of a large-scale study on well-being and suicide carried out on 14 high-schools from Alicante (Spain). The study was approved by the Ethics Committee of the University of Alicante (UA-2015-10-13), and parents provided consent to the participation of their children prior to data collection. Students aged 11 to 19 years old from 14 public secondary schools, who assented to participate anonymously, completed a battery of questionnaires online. The distribution and completion of questionnaires were overseen by research assistants during the second and third trimesters of the 2015/2016 academic year, and the process took from 60 to 70 minutes. 

#### Participants Recruitment

**Figure 1:**
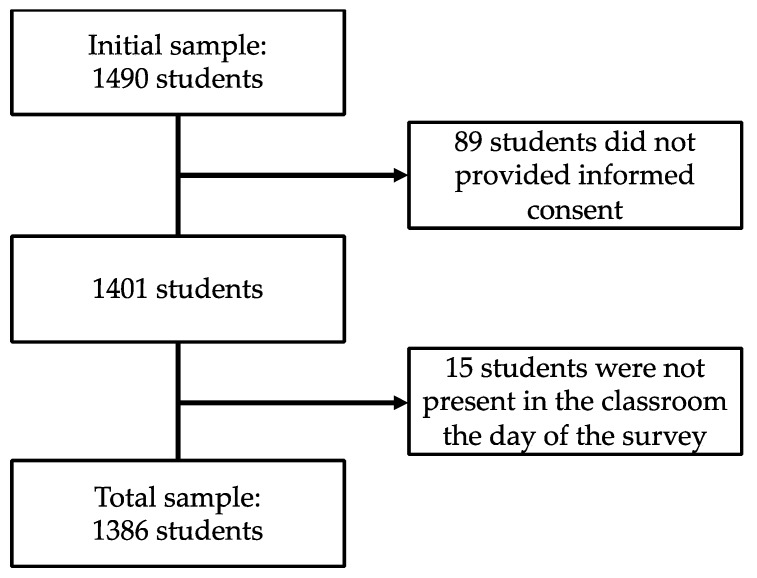
Flow-diagram of participants recruitment.

Firstly, parents were informed about the aims of the research in the first meeting of the academic year, requesting their collaboration in the study. After this initial meeting with parents, each student was provided with a letter explaining the aims and procedure of the research, along with a consent form to be signed by parents. The parents of 1490 students were contacted; of those, 89 did not return the consent form signed (6%). From a sample of 1401 students that provided consent to participate in the study, 1386 completed the questionnaires (99%). Only 15 students that provided consent did not participate because they were not present in the classroom the day of the survey (Figure 1).

The total sample was composed of 1386 adolescents (49.6% females; 50.4% males) from the province of Alicante (Valencian Community) in Spain; ranging in age from 11 to 19 years (M = 13.42, SD = 1.36). Inclusion criteria for the students were: (1) being present in the classroom on the day of the survey, (2) being able to read and complete the questionnaires on their own, and (3) presenting an informed consent form signed by their parents or legal guardians allowing their participation. Participants were only retained in the final sample if they had completed all questionnaires concerning the primary dependent measures of family function, school climate, depression, anxiety, and suicidal behavior. After obtaining the results regarding the evaluated variables, the research team gave feedback to school leaders about obtained global results regarding the prevalence of suicidal behavior in the students, in order to identify the necessity of the implementation of a school protocol to reduce and prevent suicidal behavior.

### 2.3. Statistical Analyses

Chi-square statistics were employed to identify possible differences by sex and age of participants in suicidal behavior. Pearson’s correlations were used to analyze the relationships between family function, school climate, depression, anxiety, and suicidal behavior. Hierarchical linear regression analysis was used to determine the predictive value of family function, school climate, depression, and anxiety for suicidal behavior. The indirect effects of family function and school climate on suicidal behavior through the multiple mediating effects of depression and anxiety were tested by bootstrapping. This is a non-parametric resampling procedure that can be used to test the significance of hypothesized mediation models [34]. For this, we used the PROCESS macro provided by Hayes [34], performing the resampling method (bias-corrected and accelerated) with 10,000 samples and calculating 95% confidence intervals. These analyses allowed a comparison of mediating effects between the multiple mediators included in the model. All statistical analyses were performed using IBM SPSS, Statistics for Windows, version 24.0, considering any *p* < 0.05 as significant. 

## 3. Results

### 3.1. Characterization of Suicide Behavior in the Study Sample

As seen in Table 1, 8.2% of the adolescents showed suicide ideation, 5.2% had planned to commit suicide, and 3.7% had a history of suicide attempts. Regarding frequency, 6.5% had thought about suicide in the past 12 months once, 2.6% twice, 2.2% three or four times, and 1.9% five or more times. Concerning suicide attempts, 5.1% had made a suicide attempt once and 5.3% more than once. Finally, concerning the likelihood of suicidal behavior in the future, 2.1% of the adolescents declared that it would be likely, 0.9% that it would be rather likely, and 1.6% that it would be very likely. Possible differences between sex and age groups in suicidal behavior were evaluated. No differences were found by sex in any of the evaluated subscales: lifetime suicide ideation and/or suicide attempts (χ² = 3.525, *p* = 0.317), frequency of suicidal ideation over the past 12 months (χ² = 5.820, *p* = 0.213), threat of suicide attempt (χ² = 4.593, *p* = 0.101), and self-reported likelihood of suicidal behavior in the future (χ² = 7.029, *p* =0.318). As in the case of gender, no differences were found between age groups in any of the suicidal behavior subscales: lifetime suicide ideation and/or suicide attempts (χ² = 3.560, *p* = 0.313), frequency of suicidal ideation over the past 12 months (χ² = 1.485, *p* = 0.829), threat of suicide attempt (χ² = 0.725, *p* = 0.696) and self-reported likelihood of suicidal behavior in the future (χ² = 8.025, *p* = 0.236) (Table 1).

### 3.2. Relationships between Family Function, School Climate, Depression, Anxiety, and Suicidal Behavior

The full pattern of correlations is summarised in Table 2. All of the family function dimensions (strengths and adaptability, overwhelmed by difficulties, and disrupted communication) were negatively correlated with school climate and positively correlated with depression, anxiety, and suicidal behavior (*p* < 0.001 in all cases). In the case of school climate, all of its dimensions were negatively related to depression, anxiety, and suicidal behavior (*p* < 0.001 in all cases). Finally, depression, anxiety, and suicidal behavior were positively associated (*p* < 0.001 in all cases) (Table 2).

### 3.3. Predictive Value of Family Function and School Climate for Suicidal Behavior

With the aim of analyzing the predictive value of family function, school climate, depression, and anxiety for suicidal behavior, hierarchical regression analyses were performed. For controlling the possible confounding effects of age and sex, these two factors were included in the first step. Then, family function and school climate were included in the second step, and depression and anxiety in the third. When age and sex were included, both were found to be significant predictors, explaining 0.8% of the variance in suicidal behavior. Subsequently, when family function and school climate were added to the model, only sex remained significant, and both family functioning and school climate were found to be significant predictors. In this step, the model predicted 7.1% of the variance in suicidal behavior. Finally, in the third step, when depression and anxiety were included, only depression was a significant predictor; age, sex, family function, school climate, and anxiety became non-significant. The final model explained 32.8% of the variance in suicidal behavior (Table 3).

### 3.4. Multiple Mediating Effects of Depression and Anxiety on the Relationship of Family Function and School Climate with Suicidal Behavior

**Figure 2:**
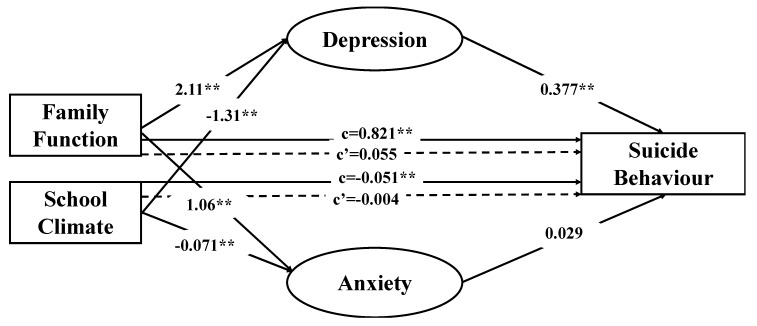
Results of multiple mediation analyses exploring the mediating effect of anxiety and depression on the association of family function and school climate with suicide ideation (** *p* < 0.01). Values presented are standardized regression coefficients. “c” indirect effect; and ‘‘c´” direct effect. Dashed lines represent non-significant effects.

In order to analyze the possible mediating effect of depression and anxiety on the association of family function and school climate with suicidal behavior, mediation analyses were performed employing a bootstrapping method. Analysing the mediating effects of depression and anxiety on the association between family function and suicidal behavior, the indirect effect of depression was estimated to lie between 0.593 and 1.04, and that of anxiety between −0.083 and 0.016, while the total indirect effect estimated in the model lay between 0.572 and 0.989, with 95% confidence in all cases. Taking into account that a mediating effect is significant when the 95% confidence interval does not span zero, it can be concluded that family function has a significant indirect effect on suicidal behavior, but only through the effect of depression as a significant mediator, unlike anxiety. In the association between school climate and suicidal behavior, depression was found to be a significant mediator, with an estimated effect of −0.064 to −0.035, and anxiety was not significant, with an estimated effect of −0.000 to 0.005, while the total indirect effect was estimated to be −0.061 to −0.034, with 95% confidence in all cases. As in the previously tested model, the total indirect effect was significant, but with only depression as a significant mediator. For both models, age and sex were included as covariates in order to control for their possible confounding effects. The magnitudes of the mediating effects of depression and anxiety have not been compared due to the lack of significance of anxiety as a mediator. Standardized regression coefficients and direct and indirect effects are represented in Figure 2.

## 4. Discussion

The present study aims to characterize suicidal behavior among Spanish adolescents, analysing the protective effects of family and school, on this phenomenon. Secondly, the current study examined the possible mediating effects of depression and anxiety on the relationship of family and school with suicidal behavior, comparing the possible differences in their mediating role.

Regarding the prevalence of suicidal behavior, the results of the present study are very similar to those obtained in previous research, both from national and international perspectives [1,2,3]. In this study, suicide ideation was the most prevalent suicide behavior (8.2%), followed by suicide planning (5.2%) and suicide attempts (3.7%), that is, the prevalence decreased with the severity of suicidal behavior. On the other hand, taking into account that a transition from suicide ideation to suicide attempt is very common during adolescence [35], eight out of every hundred adolescents in the sample evaluated can be considered to be at a severe risk of suicide. However, although previous studies have found differences in suicidal behavior by sex, showing females to have a higher risk of suicide attempts and males have a higher risk of suicidal death [36], no differences have emerged in the present study. Similarly, differences in suicidal behavior by age have not been found in the present study. Generally, as expected, these results reveal a worrisome pattern that needs to be addressed with the highest priority by the public health system.

Given the need to identify community factors that could be associated with suicidal behavior, the roles of family function and school climate on this pattern of behavior were evaluated. The results obtained indicate that lower rates of suicide behavior are significantly associated with adaptive family function and a positive school climate. According to previous studies, when family function is maladaptive, adolescents exhibit greater feelings of loneliness [15] and reduced perceptions about feeling cared for, wanted, and loved by family members, therefore increasing suicidal behavior [37]. The mechanisms underlying the effects of school climate on suicidal behavior are very similar to those described for family function, and are based on the perception of school connection and caring relationships with peers, teachers, and school staff [38,39]. Feeling socially disconnected and unsupported, both by family and school, gives rise to the phenomenon of thwarted belongingness, one of the main precursors of suicidal behavior during adolescence, according to the interpersonal theory of suicide [37,40]. This mechanism would explain the results obtained. Nevertheless, considering the results from the mediation analyses, this relationship seems not to be direct; rather, it is fully mediated by depression. The link between depression and suicide during adolescence has been extensively explored in previous research [23,41] and is characterized by various psychological, biological, and social mechanisms [42]. Indeed, as there is evidence that depression is a main determinant of suicidal behavior, the worrisome increase in rates of depression among adolescents worldwide [43] aggravates the severe public health concern about suicide.

Despite previous studies having found that anxiety could also play a main role in the development of suicide in adolescents [24,44], unlike depression, this variable did not remain significant in our analysis. Surprisingly, and contrary to the results obtained in some previous studies [24], anxiety did not mediate the relationship between the two health protective factors evaluated and suicidal behavior. However, at the same time, our results are consistent with previous research in which no association between anxiety and suicide during childhood and adolescence was found [24,45,46,47]. For example, in the study conducted by D’Eramo et al. [46], no differences in any anxiety disorder were found between non-suicidal, suicidal ideators, suicidal attempters, and multiple suicidal attempter youth. Similarly, studies that evaluated anxiety symptoms in samples of adolescents dead by suicide did not find significant differences in the rate of anxiety disorders between those who died by suicide and either suicide attempters or control individuals [48]. However, as has been found in the present research, other studies that found no association between anxiety and suicide in this population, did find a positive relationship between suicide and affective disorders such as depression [49,50]. These findings, similar to those obtained in the present study, are particularly important in this context, as they imply that clinicians and researchers should focus their attention on the mechanisms that underlie the link between suicide and affective disorders, mainly depression, as this may be the single main determinant of suicidal behavior, rather than the effects of anxiety. In this regard, based on the previous obtained results, it seems that affective disorders could have a stronger link with suicidal behavior in comparison to anxiety, probably due to the higher severity of the former and the high comorbidity of these type of disorders with other psychiatric conditions. Thus, a recent review evaluating the association between anxiety and suicide pointed out the necessity of controlling the effects of other comorbid psychiatric conditions that could have higher effects on suicide, mainly depression [24], as we have done in the present study. 

Although the present study provides meaningful information about suicidal behavior in Spanish adolescents, some limitations should be taken into account. Firstly, the cross-sectional nature of the study means that causality cannot be established, and the obtained results should be considered cautiously. Furthermore, both family function and school climate were self-reported, and the self-perception of some adolescents could be biased. However, the sample size is adequate and representative of the adolescent population to establish significant results that could help researchers improve their understanding of suicidal behavior during adolescence. In this regard, future longitudinal research should be developed in order to identify and evaluate the causal effect of family dysfunction and negative school climates on several characteristics of suicidal behavior. Further, the differential effects of psychological symptoms and mental disorders on suicidal behavior should also be analyzed deeply, with the aim to have better knowledge about the complex relationships between these phenomena. 

## 5. Conclusions

Recalling that suicide is a leading cause of death, and depression is the number one cause of illness and disability in adolescents [51], we are facing a serious public health problem, with severe consequences. To our knowledge, this is the first study seeking to compare the differential mediating effects of depression and anxiety on the previously described association of suicidal behavior with community factors, namely, family and school. Mental health interventions promoting adaptive family function and a positive school climate are necessary in order to prevent depression and suicidal behavior among adolescents. This implies that future studies should develop and evaluate the effectiveness of public health programs seeking to reduce suicide behavior by promoting adaptive family function and a positive school climate, and thereby, decrease depression. 

## Figures and Tables

**Table 1:** Characterization of suicidal behavior of the total sample (*n* = 1386) based on Suicidal Behaviors Questionnaire (SBQ-R) scores.

**Lifetime Suicide Ideation and/or Suicide Attempts**								
		Non-Suicidal Subgroup	Suicide Risk Ideation Subgroup	Suicide Plan Subgroup	Suicide Attempt Subgroup			
Total sample *n* = 1386		1149 (82.9%)	114 (8.2%)	72 (5.2%)	51 (3.7%)			
Sex	Female *n* = 688	558 (81.1%)	61 (8.9%)	39 (5.7%)	30 (4.4%)			
	Male *n* = 698	591 (84.7%)	53 (7.6%)	33 (4.7%)	21 (3%)			
Age	11–13 *n* = 666	556 (83.5%)	57 (8.6%)	35 (5.3%)	18 (2.7%)			
	14–19 *n* = 720	593 (82.4%)	57 (7.9%)	37 (5.1%)	33 (4.6%)			
**Frequency of Suicide Ideation over the Past 12 Months**								
		Never	Rarely (Once)	Sometimes (Twice)	Often (3-4 Times)	Very Often (5 or More Times)		
Total sample *n* = 1386		1203 (86.8%)	90 (6.5%)	36 (2.6%)	30 (2.2%)	27 (1.9%)		
Sex	Female *n* = 688	587 (85.3%)	49 (7.1%)	18 (2.6%)	15 (2.2%)	19 (2.8%)		
	Male *n* = 698	616 (88.3%)	41 (5.9%)	18 (2.6%)	15 (2.1%)	8 (1.1%)		
Age	11–13 *n* = 666	585 (87.8%)	40 (6%)	17 (2.6%)	13 (2%)	11 (1.7%)		
	14–19 *n* = 720	618 (85.8%)	50 (6.9%)	19 (2.6%)	17 (2.4%)	16 (2.2%)		
**Threat of Suicide Attempt**								
		No	Yes, on One Occasion	Yes, More Than Once				
Total sample *n* = 1386		1242 (89.6%)	71 (5.1%)	73 (5.3%)				
Sex	Female *n* = 688	605 (87.9%)	43 (6.3%)	40 (5.8%)				
	Male *n* = 698	637 (91.3%)	28 (4%)	33 (4.7%)				
Age	11–13 *n* = 666	598 (89.8%)	36 (5.4%)	32 (4.8%)				
	14–19 *n* = 720	644 (89.4%)	35 (4.9%)	41 (5.7%)				
**Self-Reported Likelihood of Suicidal Behavior in the Future**								
		Never	No Chance at all	Rather Unlikely	Unlikely	Likely	Rather Likely	Very Likely
Total sample *n* = 1386		1084 (78.2%)	143 (10.3%)	65 (4.7%)	31 (2.2%)	29 (2.1%)	12 (0.9%)	22 (1.6%)
Sex	Female *n* = 688	522 (75.9%)	75 (10.9%)	34 (4.9%)	17 (2.5%)	19 (2.8%)	8 (1.2%)	13 (1.9%)
	Male *n* = 698	562 (80.5%)	68 (9.7%)	31 (4.4%)	14 (2%)	10 (1.4%)	4 (0.6%)	9 (1.3%)
Age	11–13 *n* = 666	536 (80.5%)	65 (9.8%)	28 (4.2%)	12 (1.8%)	10 (1.5%)	3 (0.5%)	12 (1.8%)
	14–19 *n* = 720	548 (76.1%)	78 (10.8%)	37 (5.1%)	19 (2.6%)	19 (2.6%)	9 (1.3%)	10 (1.4%)

**Table 2:** Patterns of correlations between family function, school climate, depression, anxiety, and suicidal behavior.

		1	2	3	4	5	6	7	8	9	10	11	12	13
**Family Function**	**1.Strengths and Adaptability**	-	0.248 **	0.173 **	0.585 **	−0.249 **	−0.296 **	−0.197 **	−0.184 **	−0.262 **	−0.326 **	0.310 **	0.179 **	0.223 **
	**2.Overwhelmed by Difficulties**		-	0.649 **	0.846 **	−0.209 **	−0.252 **	−0.241 **	−0.142 **	−0.265 **	−0.299 **	0.293 **	0.152 **	0.167 **
	**3.Disrupted Communication**			-	0.833 **	−0.200 **	−0.172 **	−0.196 **	−0.088 **	−0.200 **	−0.237 **	0.196 **	0.109 **	0.106 **
	**4.Total Family Function**				-	−0.285 **	−0.309 **	−0.277 **	−0.176 **	−0.315 **	−0.373 **	0.345 **	0.189 **	0.211 **
**School Climate**	**5.Satisfaction**					-	0.428 **	0.467 **	0.532 **	0.568 **	0.865 **	−0.265 **	−0.171 **	−0.146 **
	**6.Sense of Belonging**						-	0.261 **	0.264 **	0.372 **	0.652 **	−0.252 **	−0.164 **	−0.189 **
	**7.Integration**							-	0.566 **	0.412 **	0.656 **	−0.326 **	−0.214 **	−0.229 **
	**8.Cooperation**								-	0.493 **	0.708 **	−0.286 **	−0.189 **	−0.173 **
	**9. Communication between** **Family and School**									-	0.785 **	−0.230 **	−0.112 **	−0.118 **
	**10.Total School Climate**										-	−0.352 **	−0.218 **	−0.216 **
	**11.Depression**											-	0.599 **	0.574 **
	**12.Anxiety**												-	0.316
	**13.Suicide Behavior**													-

** *p* < 0.001.

**Table 3:** Regression analyses of sociodemographic variables (age, sex), family function, school climate, depression, and anxiety as predictors of suicidal behavior.

	Step 1			Step 2			Step 3		
	β	R2	ΔR2	β	R2	ΔR2	β	R2	ΔR2
**Age**	0.066 *			0.022			−0.006		
**Sex**	−0.061 *			−0.066 *			0.001		
F(2,1379) = 5.313, *p* = 0.005		0.008	0.008 **						
**Total Family Functioning**				0.157 **			0.010		
**Total School Climate**				−0.152 **			−0.015		
F(4,1379) = 26.327, *p* = 0.0001					0.071	0.063 **			
**Depression**							0.591 **		
**Anxiety**							−0.042		
F(6,1379) = 113.226, *p* = 0.0001								0.328	0.260 **

* *p* < 0.05; ** *p* < 0.01.
